# *S*‐allyl‐l‐cysteine (SAC) protects hepatocytes from alcohol‐induced apoptosis

**DOI:** 10.1002/2211-5463.12684

**Published:** 2019-06-17

**Authors:** Peng Chen, Mingdao Hu, Feng Liu, Henghai Yu, Chen Chen

**Affiliations:** ^1^ Department of Hepatopancreatobiliary Surgery the Second Affiliated Hospital of Kunming Medical University China; ^2^ Department of Ophthalmology the Second People's Hospital of Yunnan Province Kunming China

**Keywords:** alcohol, apoptosis, hepatocyte, oxidative stress, *S*‐allyl‐l‐cysteine

## Abstract

Hepatocyte apoptosis is frequently observed in alcohol‐related liver disease (ARLD), which ranks among the 30 leading causes of death worldwide. In the current study, we explored the impact of *S*‐allyl‐l‐cysteine (SAC), an organosulfur component of garlic, on hepatocyte apoptosis induced by alcohol. Rat liver (BRL‐3A) cells were challenged by ethanol with or without SAC treatment. Cell death/viability, reactive oxygen species (ROS) generation, mitochondrial Cytochrome C release, and caspase 3 activity were then examined. We found that ethanol remarkably induced apoptosis of hepatocytes, while SAC treatment rescued ethanol‐induced hepatocyte injury, as demonstrated by cell counting kit‐8 (CCK8) assay, TUNEL assay, and annexin V/PI staining assay. Ethanol evoked ROS generation in BRL‐3A cells, and this was abated by SAC pretreatment, as indicated by 2′,7′‐dichlorofluorescin diacetate (DCFDA) staining assay. Moreover, ethanol suppressed cellular anti‐apoptotic protein B‐cell lymphoma‐2 (Bcl‐2) expression, increased pro‐apoptotic protein Bcl‐2‐associated X protein (Bax) expression, induced mitochondrial Cytochrome C release, and activated the caspase 3‐dependent apoptosis pathway in BRL‐3A cells. SAC was sufficient to abolish all these changes induced by ethanol, thereby revealing the molecular mechanisms underlying its protective effects. In conclusion, SAC protects hepatocytes from ethanol‐induced apoptosis and may be suitable for use as a novel anti‐apoptotic agent for treating ARLD.

**Abbreviations**
AGE: aged garlic extract
ARLD: alcohol‐related liver disease
Bax: Bcl‐2‐associated X protein
Bcl‐2: B‐cell lymphoma‐2
Bip: binding immunoglobulin protein
CCK8: cell counting kit‐8
cGMP: cyclic guanosine monophosphate
CHOP: C/EBP‐homologous protein
CYP2E1: cytochrome P450 2E1
DAPI: 4′,6‐diamidino‐2‐phenylindole
DCFDA: 2′,7′‐dichlorofluorescin diacetate
ER: endoplasmic reticulum
FITC: fluorescein isothiocyanate
MMP: mitochondrial membrane potential
NADH: nicotinamide adenine dinucleotide
NO: nitric oxide
Nrf2: nuclear factor erythroid‐2‐related factor 2
PFA: paraformaldehyde
PIC: protease inhibitor cocktail
PI: propidium iodide
PMSF: phenylmethylsulfonyl fluoride
RIPA: radioimmunoprecipitation assay
ROS: reactive oxygen species
RT: room temperature
SAC: *S*‐allyl‐l‐cysteine
TUNEL: terminal deoxynucleotidyl transferase‐mediated uridine 5′‐triphosphate‐biotin nick end labeling

Alcohol‐related liver disease (ARLD) ranks among the 30 leading causes of death worldwide 1. At present, alcohol is believed to be implicated in up to 50% of mortality caused by cirrhosis 1. ARLD consists of a spectrum of alcohol‐induced liver pathologies, including steatosis, alcoholic steatohepatitis, progressive fibrosis, and cirrhosis 2. Among these pathological processes, hepatocyte injury, featured by increased hepatocyte apoptosis, is a striking characteristic. Excessive hepatocyte apoptosis can eventually lead to liver dysfunction, fibrosis, cirrhosis, and tumorigenesis 3, 4.

Alcohol consumption induces generation of ROS in the liver, the organ where alcohol is metabolized. To date, several ethanol metabolism pathways have been shown to produce reactive intermediates that affect cellular antioxidant system in hepatocytes 5, 6. Ethanol metabolism catalyzed by alcohol dehydrogenase produces acetaldehyde and nicotinamide adenine dinucleotide (NADH), as well as free radicals 6. NADH interferes with the mitochondrial electron transfer system, while the formation of acetaldehyde causes mitochondrial damage, both of which lead to more ROS production 7, 8, 9. The cytochrome P450 2E1 (CYP2E1) is also a significant catalyst of ethanol metabolism. It metabolizes ethanol to acetaldehyde and 1‐hydroxyethyl radical, which produces ROS 10. When ROS accumulates to a critical degree, mitochondrial function is damaged, which results in Cytochrome C release and succedent activation of the caspase‐dependent apoptosis cascade 11.

Garlic has been utilized both for flavor and for medicinal purposes for centuries 12. Aged garlic extract (AGE), a product from long‐term (10 to 20 months) extraction of raw garlic, exhibits hypolipidemic, neuroprotective, and cancer preventive effects 13, 14, 15, 16, 17. *S*‐allyl‐l‐cysteine (SAC) is a biological active organosulfur component of garlic, and the most abundant and active ingredient in AGE. At present, AGE and SAC have been studied extensively, and their efficacy has been attributed to their potent antioxidant functions 12. In human placenta trophoblast cells (TEV‐1) and placental explants, SAC significantly decreased H_2_O_2_‐induced ROS production, restored cellular nitric oxide (NO) and cGMP level, and was assumed to be a potential remedy for preeclampsia 18. In primary neurons, SAC activated the nuclear factor erythroid‐2‐related factor 2 (Nrf2) and Nrf2‐dependent antioxidative system, and protected cells from oxidative injury caused by oxygen and glucose deprivation 19. Moreover, in neuronal pheochromocytoma (PC12) cells insulted with the complex I inhibitor rotenone, SAC increased mitochondrial membrane potential (MMP), thus improved mitochondrial function 20. These findings provide solid evidence for the antioxidative properties of AGE and SAC.

In the present study, we investigate whether SAC protects ethanol‐insulted hepatocytes from apoptosis, and explore the potential mechanism.

## Materials and methods

### Chemicals

Ethanol absolute (≥99.7%) was purchased from Sinopharm Chemical Reagent Ltd (China). SAC (≥98%) was purchased from Sigma‐Aldrich (USA). SAC was dissolved in sterile‐filtered phosphate‐buffered saline (PBS) and stored at ‐20°C. The concentration of the stock solution was 50 mmol·L^−1^.

### Cell culture

The present study employed rat liver (BRL‐3A) cells as the object of the research. BRL‐3A cells were purchased from Kunming Cell Bank of the Chinese Academy of Sciences (CAS) and maintained in DMEM/F‐12 50/50 mix medium (Gibco/Invitrogen, Carlsbad, CA, USA) supplied with 10% fetal bovine serum (Hyclone, Logan, UT, USA) and 1% antibiotic/antimycotic solution (Biological Industries, Beit Haemek, Israel). Cells were subcultured when the confluence reaches 80%, and generations between 10 and 25 were used for experiments.

### CCK‐8 assay

Viability of BRL‐3A cells was determined by CCK‐8 assay (Dojindo Laboratories, Kumamoto, Japan). Cells were seeded in 96‐well microplates, and after treatment, medium was changed and CCK‐8 reagent was added (10 μL each well). The plates were incubated at 37 °C for 2 h before absorbance was determined at 450 nm by a microplate reader (Spectra Max 190, Molecular Devices, San Jose, CA, USA).

### TUNEL assay

Cell apoptosis was detected using TUNEL assay (In Situ Cell Death Detection Fluorescein Kit, Roche Diagnostics, Indianapolis, IN, USA) as previously described 21. After treatment, cells on coverslips were fixed with freshly prepared 4% paraformaldehyde (PFA) at room temperature (RT) for 1 h, and permeabilized with 0.1% citrate buffer containing 0.1% Triton X‐100 (2 min, on ice). Then, cells were rinsed with PBS and incubated at 37 C for 1 h in TUNEL reaction mix composed of label solution and enzyme solution. After extensive wash, coverslips were mounted to a slide and examined under a fluorescence microscope. The mounting medium (Vector Laboratories, Burlingame, CA, USA) contains 4′,6‐diamidino‐2‐phenylindole (DAPI) for cell nuclei staining.

### Annexin V and propidium iodide assay

When treatment completed, cells were digested with trypsin, centrifuged, resuspended, and incubated with fluorescein isothiocyanate (FITC) annexin V and PI (BD Pharmingen, USA) for 15 min at RT. Cell apoptosis rate was detected by a CyFlow Space flow cytometer (Partec, Munster, Germany).

### Detection of ROS generation in BRL‐3A cells

After treatment, cells on coverslips were incubated with 5 μm 2′,7′‐dichlorofluorescin diacetate (DCFDA, Sigma‐Aldrich, USA) for 30 min, and then, pictures were taken using a fluorescence microscope. For quantification of fluorescence, cells were subcultured in 6‐well plates. When treatment completed, cells were incubated with 5 μm DCFDA for 30 min, then washed, digested and collected, and fluorescence was determined using a CyFlow Space flow cytometer (Partec) with wavelength of excitation at 485 nm and emission at 525 nm.

### Western blotting

Western blot analysis was performed as described previously 22. In brief, BRL‐3A cells were lysed with radioimmunoprecipitation assay (RIPA) buffer containing protease inhibitor cocktail (PIC) and phenylmethylsulfonyl fluoride (PMSF) (Beijing ComWin Biotech, Beijing, China) to harvest total cellular proteins. For Cytochrome C detection, cellular mitochondria were separated from cytosol using a Cell Mitochondria Isolation Kit (Beyotime, Shanghai, China). Protein concentration was determined with a bicinchoninic acid kit (Beijing ComWin Biotech). Twenty micrograms of protein was separated by SDS/PAGE gel and electro‐transferred to nitrocellulose membranes. Membranes were blocked with 1% BSA and incubated at 4 °C for at least 16 h with following primary antibodies: anti‐Bax (1 : 1000; Cell Signaling Technology, Danvers, MA, USA), anti‐Bcl‐2 (1 : 1000; Abcam, Cambridge, UK), anti‐Cytochrome C (1 : 1000; Abcam), and anti‐caspase 3 (1 : 500; Proteintech, Rosemont, IL, USA). After wash, membranes were incubated with horseradish peroxidase‐conjugated secondary antibodies and developed with chemiluminescence substrate (Beijing ComWin Biotech) using Applied Biosystems (Life Technologies, Carlsbad, CA, USA). To detect loading control, membranes were stripped and β‐actin expression was blotted (anti‐β‐actin antibody, 1 : 4000, Origene, Rockville, MD, USA). All bands were analyzed by densitometry using imagej software (National Institute of Health, Bethesda, MD, USA).

### Immunocytochemistry

After treatment, cells on coverslips were fixed with 4% PFA for 20 min at RT, and permeabilized in 0.1% Triton X‐100 for 10 min. Coverslips were then blocked with normal goat serum and incubated with anti‐Cleaved caspase 3 antibody (1 : 100; Cell Signaling Technology) at 4 °C overnight. After wash with PBS, cells were incubated with Alexa Fluor^®^ 555‐conjugated secondary antibody (1 : 500; Cell Signaling Technology) at RT for 1 h. Then, coverslips were washed and mounted to a slide, and pictures were taken using a fluorescence microscope. The mounting medium (Vector Laboratories) contains DAPI for cell nuclei staining.

### Caspase 3 activity determination

Cellular caspase 3 activity was detected using a caspase 3 Activity Assay Kit (Beyotime). At the end of each treatment, BRL‐3A cells were collected and lysed. Bradford assay was employed to measure protein concentration. Then, samples were incubated with caspase 3 substrate Ac‐DEVD‐pNA at 37 °C for 2 h, and the absorbance at 405 nm was measured.

### Statistical analysis

Data were expressed as mean ± standard deviation (SD). graphpad prism 5.0 (San Diego, CA, USA) was employed to analyze the data, and to generate the graphs and bar charts. Statistical analyses were performed using one‐way ANOVA with Bonferroni's multiple comparison tests. A *P*‐value of less than 0.05 was considered to indicate a significant difference.

## Results

### SAC rescues BRL‐3A cells from alcohol‐induced apoptosis

**Figure 1:**
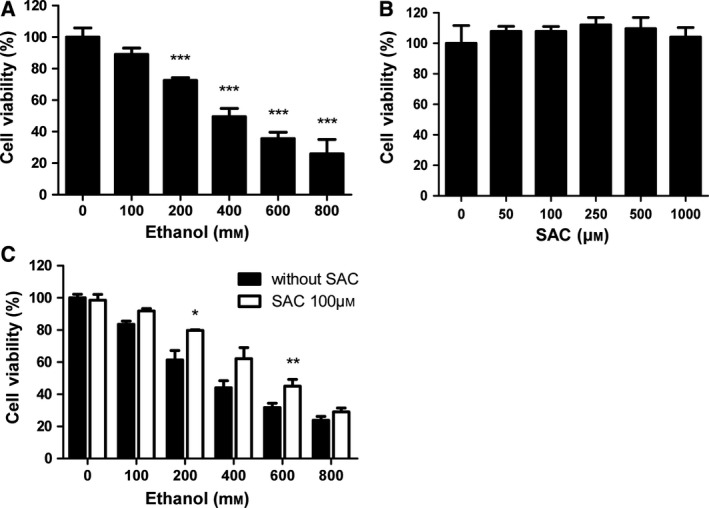
*S*‐allyl‐l‐cysteine rescues ethanol‐induced BRL‐3A cell injury. (A) BRL‐3A cells were challenged with various concentrations of ethanol for 24 h. Cell viability was detected by CCK‐8 assay (*n* = 3, ***P < 0.001 vs. Ctrl). (B) BRL‐3A cells were exposed to various concentrations of SAC for 48 h. Cell viability was measured by CCK‐8 assay (*n* = 3). (C) BRL‐3A cells were pretreated with 100** **μm SAC overnight (16 h) and then exposed to ethanol for 24 h. Cell viability was tested using CCK‐8 assay (*n* = 3, *P < 0.05, **P < 0.01 vs. cells treated with ethanol only). Data were analyzed using ANOVA with Bonferroni's multiple comparison tests. Error bars indicate SD.

To determine whether alcohol induces apoptosis in hepatocytes, we treated BRL‐3A cells with various doses of ethanol for 24 h. CCK‐8 assay was employed to detect cell viability. We found that ethanol dose‐dependently decreased BRL‐3A cell viability. At the dose of 200 mm, ethanol‐induced cell injury was statistically significant compared with untreated control. At the dose of 800 mm, cell viability was drastically decreased by 74% compared with control (Fig. 1A). To detect whether SAC *per se* has any influence on cell viability, BRL‐3A cells were exposed to various doses of SAC for 48 h. CCK‐8 assay did not show any significant change in cell viability after SAC treatment (Fig. 1B). To determine whether SAC protects BRL‐3A cells from ethanol‐induced injury, we pretreated cells with 100 μm SAC overnight before ethanol exposure. This experiment revealed that SAC significantly alleviated cell injury induced by various concentrations of ethanol (Fig. 1C).

**Figure 2:**
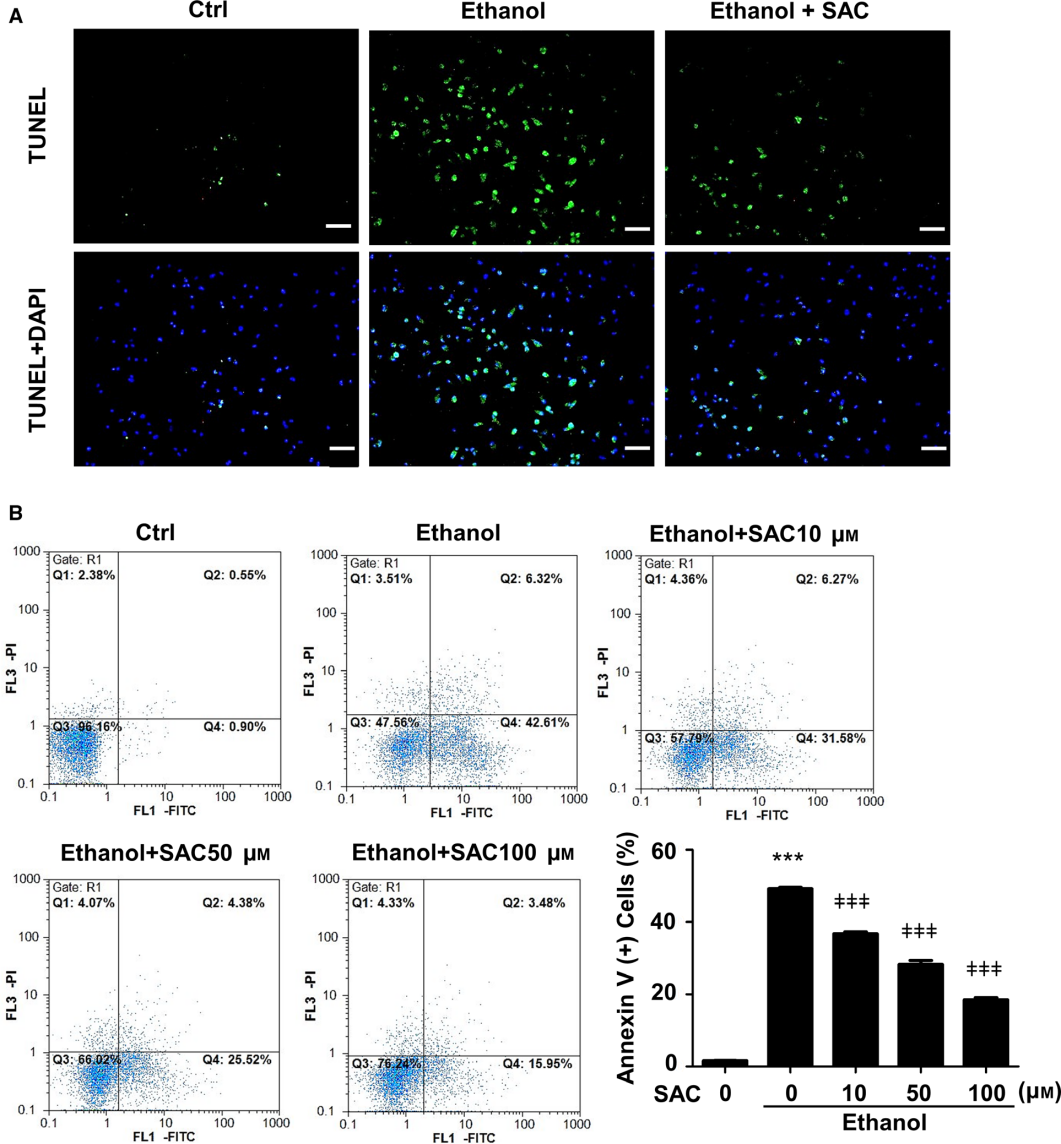
SAC protects BRL‐3A cells from ethanol‐induced apoptosis. (A) BRL‐3A cells were exposed to 500 mm ethanol for 16 h with or without pretreatment of SAC (100** **μm, 4 h). Apoptosis was measured by TUNEL staining (green: apoptotic cells; blue: cell nuclei (DAPI staining); bar: 50 μm). (B) BRL‐3A cells were exposed to 500 mm ethanol for 48 h with or without different concentrations of SAC. Apoptosis was determined using annexin V and PI assay (*n* = 3, ***P < 0.001 vs. Ctrl, ǂǂǂ*P* < 0.001 vs. cells treated with ethanol only). Data were analyzed using ANOVA with Bonferroni's multiple comparison tests. Error bars indicate SD.

TUNEL assay labels genomic DNA fragmentation and DNA damage, both of which are indications of cell injury 23. Consistent with CCK‐8 results, TUNEL staining revealed that ethanol induced remarkable cell injury, as indicated by green fluorescence (Fig. 2A). As expected, SAC reduced TUNEL‐positive cell number (Fig. 2A). The results were further confirmed with annexin V/PI staining, demonstrating that SAC dose‐dependently reduced annexin V‐positive cell number (Fig. 2B). These findings indicate that SAC protects BRL‐3A cells from ethanol‐induced cell apoptosis.

### SAC reduces ROS generation in BRL‐3A cells insulted with ethanol

**Figure 3:**
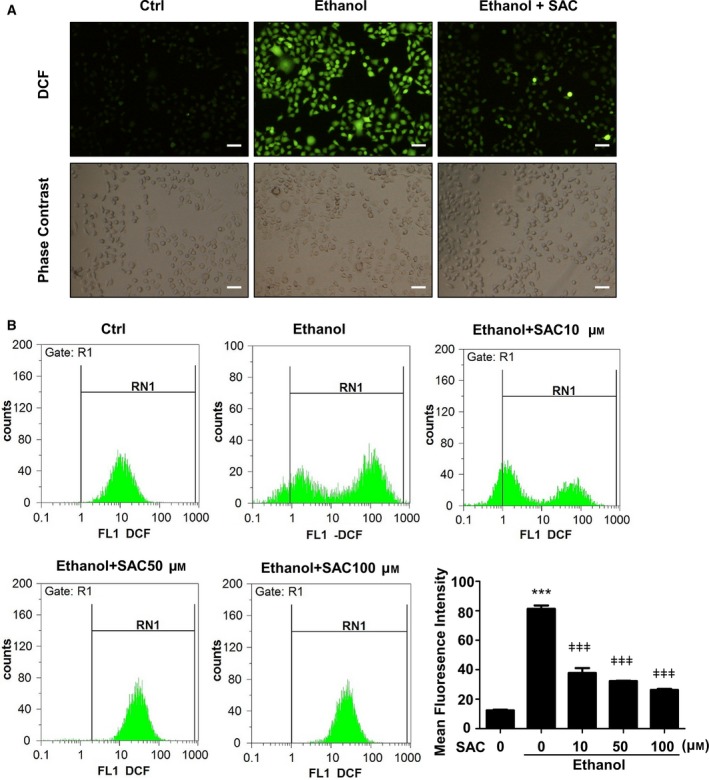
*S*‐allyl‐l‐cysteine reduces ROS generation in ethanol‐insulted BRL‐3A cells. (A) BRL‐3A cells were insulted with 500 mm ethanol for 1.5 h with or without 100 μm SAC pretreatment. ROS generation was detected by DCFDA staining (bar: 50 μm). (B) After different concentrations of SAC pretreatment overnight, BRL‐3A cells were insulted with 500 mm ethanol for 1.5 h before stained with DCFDA. ROS generation was determined by flow cytometry (*n* = 3, ***P < 0.001 vs. Ctrl, ǂǂǂ*P* < 0.001 vs. cells treated with ethanol only). Data were analyzed using ANOVA with Bonferroni's multiple comparison tests. Error bars indicate SD.

To determine whether SAC decreases ethanol‐induced ROS generation, we stained cells with DCFDA. DCFDA is converted into 2′,7′‐dichlorofluorescein (DCF) by cellular ROS, and DCF presents high fluorescence. This experiment revealed remarkable ROS generation upon ethanol treatment, which was abated by SAC (Fig. 3A). We next employed flow cytometry to quantify the fluorescence of DCF. Consistently, SAC decreased ethanol‐induced ROS generation in BRL‐3A cells (Fig. 3B), suggesting that SAC may protect cells through antioxidative mechanisms.

### Ethanol decreases Bcl‐2 and increases Bax expression, both of which are reversed by SAC

**Figure 4:**
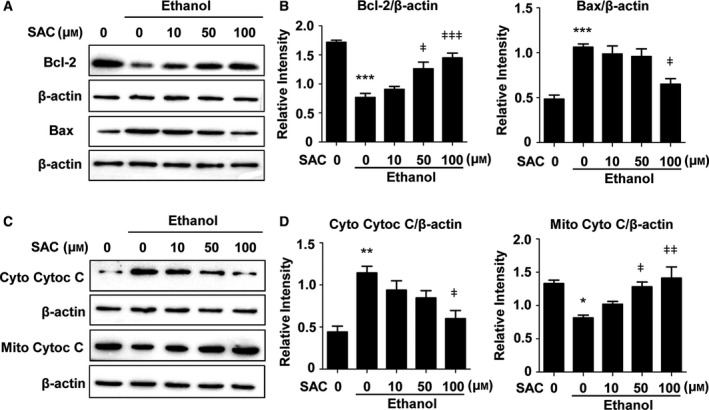
Ethanol decreases Bcl‐2 expression, increases Bax expression, and induces mitochondrial Cytochrome C releasing, all of which are abated by SAC. BRL‐3A cells were treated with various doses of SAC overnight and then challenged with 500 mm ethanol for 8 h. Bcl‐2 and Bax expression levels were measured by western blot (A) and analyzed using densitometry (B) (*n* = 3, ***P < 0.001 vs. Ctrl, ǂ*P* < 0.05, ǂǂǂ*P* < 0.001 vs. cells treated with ethanol only). Cytosolic and mitochondrial Cytochrome C levels were detected by western blot (C) and semi‐quantified by densitometry (D), respectively (*n* = 3, *P < 0.05, **P < 0.01 vs. Ctrl, ǂ*P* < 0.05, ǂǂ*P* < 0.01 vs. cells treated with ethanol only). Data were analyzed using ANOVA with Bonferroni's multiple comparison tests. Error bars indicate SD.

To further explore the mechanism by which SAC protects hepatocytes from apoptosis, we determined the protein levels of apoptosis‐related factors Bcl‐2 and Bax using western blot. We found that Bcl‐2 protein level was reduced by 55% after ethanol treatment, which was partly reversed by SAC (Fig. 4A,B). On the contrary, Bax expression increased to 2.2‐fold of the control upon ethanol treatment, which was also reversed by SAC (Fig. 4A,B).

### SAC abrogates ethanol‐induced mitochondrial Cytochrome C release

When mitochondrial function is damaged, Cytochrome C in the intermembrane space is released into the cytoplasm, which is a common incident in cell apoptosis and evokes a terminal caspase‐dependent apoptotic pathway 24, 25. To determine whether ethanol and SAC influence Cytochrome C release, we isolated cellular mitochondria from cytosol and detected mitochondrial and cytosolic Cytochrome C levels, respectively, using western blot. These experiments revealed a remarkable increase in mitochondrial Cytochrome C release, as demonstrated by a 2.6‐fold increase in the cytosolic level, and a 39% decrease in the mitochondrial level of Cytochrome C (Fig. 4C,D). With SAC treatment, Cytochrome C release was significantly reduced, as indicated by reversed levels of both cytosolic and mitochondrial Cytochrome C (Fig. 4C,D).

### SAC abolishes caspase 3 activation induced by ethanol

**Figure 5:**
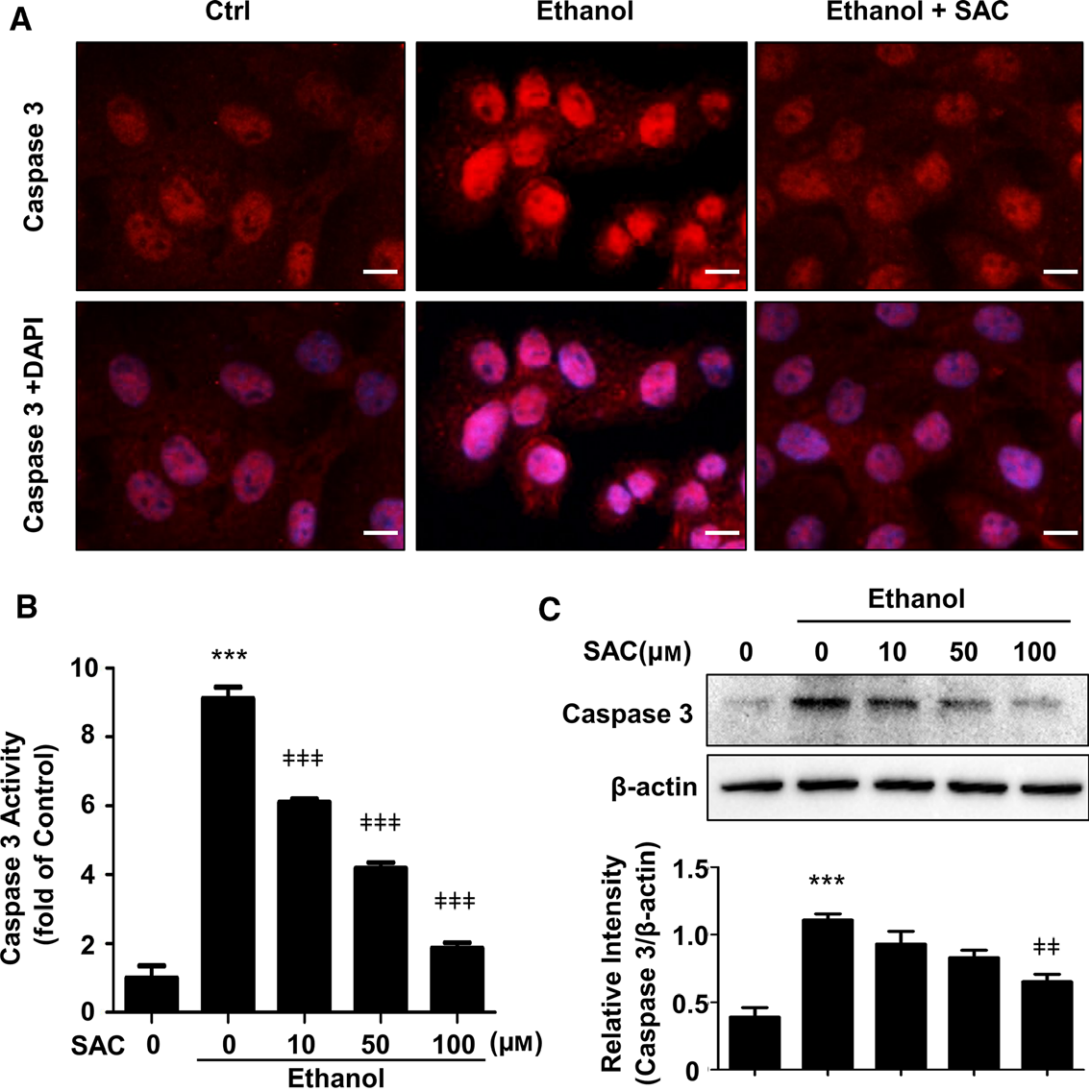
*S*‐allyl‐l‐cysteine decreases caspase 3 activation induced by ethanol. (A) BRL‐3A cells were treated with 100 μm SAC overnight before being exposed to 500 mm ethanol for 8 h. Caspase 3 activity was determined by immunocytochemistry (bar: 10 μm). (B) BRL‐3A cells were pretreated with various doses of SAC for 4 h and then challenged with 500 mm ethanol for 16 h. Caspase 3 activity was measured with caspase 3 Activity Assay Kit (***P < 0.001 vs. Ctrl, ǂǂǂ*P* < 0.001 vs. ethanol treatment without SAC). (C) BRL‐3A cells were treated with various doses of SAC overnight and then exposed to 500 mm ethanol for 8 h. Caspase 3 protein level was detected by western blot and analyzed using densitometry (*n* = 3, ***P < 0.001 vs. Ctrl, ǂǂ*P* < 0.01 vs. cells treated with ethanol only). Data were analyzed using ANOVA with Bonferroni's multiple comparison tests. Error bars indicate SD.

**Figure 6:**
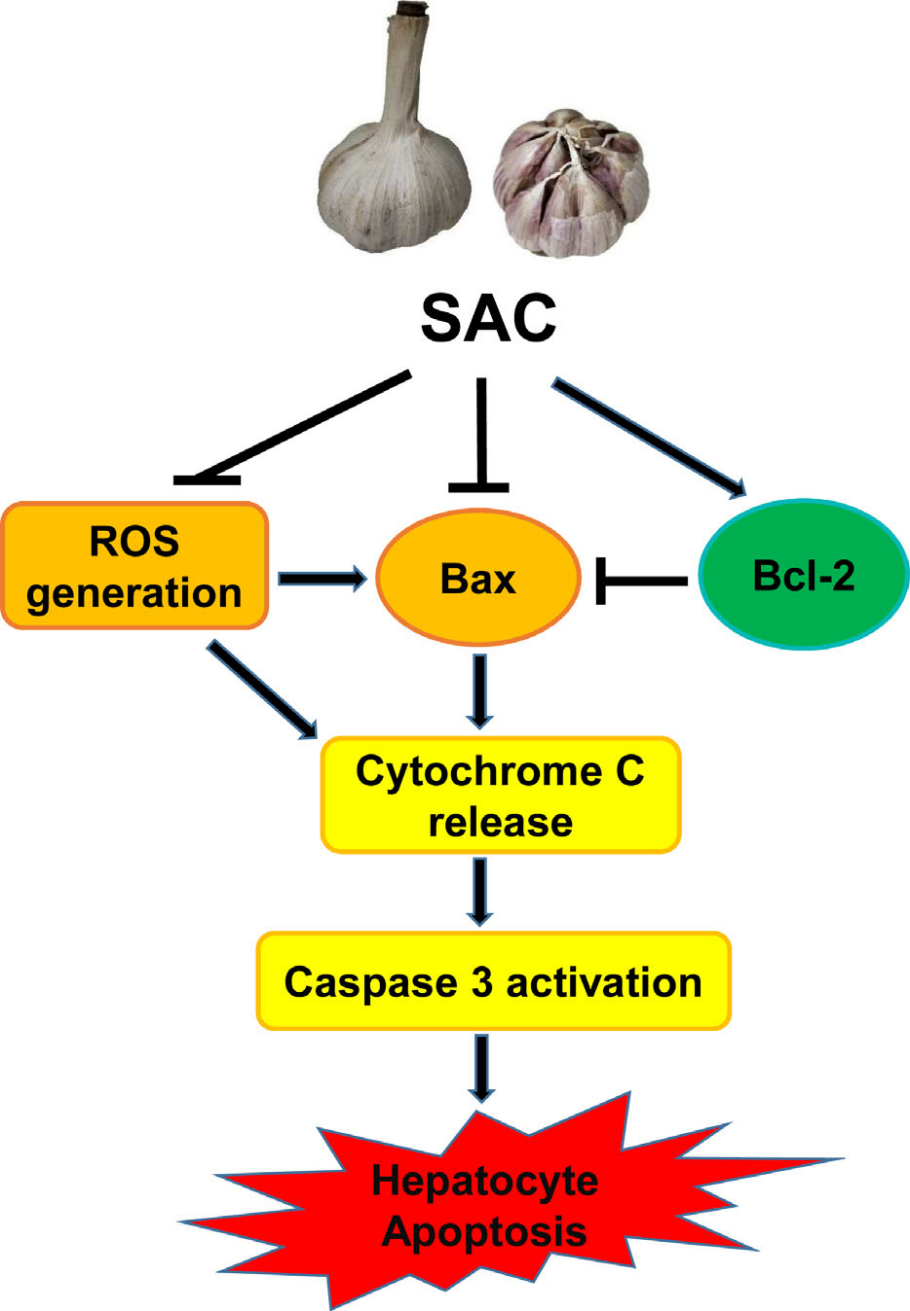
Schema of the protective effects of SAC in hepatocytes. ROS generation and Bax upregulation lead to mitochondrial Cytochrome C release and activation of Caspase‐dependent apoptosis cascade. SAC increases Bcl‐2 level, suppresses ROS generation and Bax expression, and protects hepatocytes from apoptosis.

To determine whether caspase 3 is implicated in ethanol‐induced BRL‐3A apoptosis, we detected Cleaved caspase 3 expression by immunocytochemistry. We found a notable increase of Cleaved caspase 3 level in cell nuclei, which was abolished by SAC (Fig. 5A). We next determined caspase 3 activity in BRL‐3A cells using a caspase 3 Activity Assay Kit. Consistent with immunocytochemistry, ethanol drastically increased caspase 3 activity, whereas SAC dose‐dependently abated caspase 3 activation (Fig. 5B). Finally, we employed western blotting to further confirm caspase 3 protein level. As expected, we found a 2.8‐fold increase in caspase 3 level upon ethanol treatment, which was reversed by SAC (Fig. 5C). These results provide the potential mechanisms for the cytoprotective properties of SAC. Figure 6 is a graphical representation of the mechanisms involved in the hepatoprotective functions of SAC.

## Discussion

Acute and chronic alcohol consumption induces excessive ROS production, evokes oxidative stress, and eventually leads to hepatocyte apoptosis 11. Various studies demonstrate that antioxidants attenuate liver injury caused by alcohol 26, 27, 28. Consistent with previous findings, the current study demonstrates that alcohol induces ROS generation and mitochondrial Cytochrome C release and thus triggers caspase 3‐dependent apoptosis in hepatocytes, whereas SAC abolishes all these changes induced by alcohol and alleviates hepatocyte injury.

The mechanisms of the antioxidative function of SAC include the following: clearing up of ROS and reactive nitrogen species, induction of antioxidative enzymes, suppression of prooxidant enzymes, and activation of Nrf2 signaling pathway 12. Various studies have shown that SAC scavenges hydrogen peroxide, superoxide anion, and peroxynitrite anion, and prevents lipid/protein oxidation and nitration 12, 29, 30, 31, 32. Administration of SAC in Balb/cA mice improved glutathione peroxidase and catalase function in liver and kidney, and reduced lipid oxidation in both organs 33. In a rat renal damage model, SAC suppressed the function of NADPH oxidase, which was capable of producing superoxide anion, and ameliorated hypertension and renal damage 34. Last but not the least, SAC activates the transcription factor Nrf2, which acts as a central regulator in cellular antioxidant defense. Kalayarasan *et al*. 35 reported that intraperitoneal injection of SAC increased Nrf2 expression in rat liver, which subsequently activated antioxidant and phase II enzymes to protect the hepatocytes. Shi *et al*. reported that SAC administration in wild‐type (Nrf2^+/+^) mice alleviated ischemic injury to the brain following transient middle cerebral artery occlusion. However, in Nrf2^−/−^ mice, the neuroprotective function of SAC was absent, evidence of the involvement of Nrf2 in SAC cytoprotective function 19.

In the intrinsic apoptosis pathway, the Bcl‐2 family proteins maintain the integrity of mitochondria and regulate cell fate 36. Among these proteins, anti‐apoptotic factor Bcl‐2 localizes at the mitochondria membrane and inhibits apoptosis, while Bax commits cells to death by increasing the outer mitochondrial membrane permeability, which subsequently leads to mitochondrial protein release to the cytoplasm 36, 37. In patients with ARLD, both Bcl‐2 and Bax expression were shown to be increased in liver tissue 38, 39. Moreover, in ethanol diet‐fed mice, Bcl‐2 and Bax levels were both up‐regulated compared with control 39. However, the current study demonstrates that ethanol increases Bax level and reduces Bcl‐2 level in hepatocytes. The difference between our results and literature may be attributed to different alcohol exposure modes. Our study focused on an acute exposure of hepatocytes to ethanol, which may evoke different cellular responses compared with chronic alcohol exposure, as in patients diagnosed with ARLD or mice fed with ethanol diet for weeks. In our study, acute exposure to ethanol increases Bax/Bcl‐2 ratio by inducing the former and reducing the latter and triggers remarkable apoptosis within 16 h.

The mechanisms for alcohol‐induced hepatocyte apoptosis include not only oxidative stress, but also membrane death receptor‐related pathway, endoplasmic reticulum (ER) stress, and altered proteasome function 11. These mechanisms intermix each other, and alcohol‐induced hepatocyte apoptosis may be controlled by multiple signaling pathways 11. In alcoholic hepatitis patients, Fas levels were significantly higher compared with control both in plasma 40 and in liver tissue 41, indicating a possible role of Fas/FasL‐mediated apoptosis in alcoholic hepatitis. In liver tissue obtained from patients with chronic ARLD, significant upregulation of ER chaperone binding immunoglobulin protein (Bip) was observed. Furthermore, the expression of ER stress‐associated pro‐apoptotic factor C/EBP‐homologous protein (CHOP) was also elevated 42. Extensive studies have shown that alcohol consumption inhibits proteasome function, which subsequently induces DNA fragmentation and cell death in liver cells 43, 44, 45. Oxidative stress, ER stress, death receptor‐mediated cascade, altered proteasome function, and other mechanisms may work synergically, leading to mitochondrial dysfunction, Caspase activation, and eventually cell apoptosis. Whether SAC exerts any effects on these diverse signaling pathways still remains to be elucidated.

Taken together, our results demonstrate that SAC, an active organosulfur compound in garlic, scavenges cellular ROS, alleviates ethanol‐induced mitochondria dysfunction, and protects hepatocytes from apoptosis. SAC may be a potential candidate for the pharmacological therapy of ARLD.

## Conflict of interest

The authors declare no conflict of interest.

## Author contributions

PC and CC conceived and designed the project, interpreted the data, and wrote the paper. PC, MH, FL, and HY performed experiments and analyzed the data. MH and HY provided critical comments on the manuscript.
